# Effectiveness of Platelet-Rich Plasma in the Treatment of Androgenic Alopecia: A Meta-Analysis

**DOI:** 10.1007/s00266-023-03603-9

**Published:** 2023-08-29

**Authors:** Meijia Li, Kaipan Qu, Qiang Lei, Mingrui Chen, Donghui Bian

**Affiliations:** 1 Department of Burn and Plastic Surgery, The 960th Hospital of the People’s Liberation Army, No. 25 Shifan Road, Jinan, 250001 China; 2 Department of Burn and Plastic Surgery, Shandong Provincial Third Hospital, Jinan, China

**Keywords:** Platelet-rich plasma, PRP, Androgenic alopecia, AGA, Injection therapy

## Abstract

**Background:**

Androgenetic alopecia (AGA) is a common yet difficult-to-treat condition, which is an important psychosocial problem. Platelet-rich plasma (PRP) therapy has been considered as a promising treatment for AGA. However, the current evidence on the efficacy of PRP for treating AGA is still controversial. This study evaluated the efficacy of PRP monotherapy in the treatment of AGA.

**Methods:**

We searched PubMed, Embase, Cochrane Library and Web of Science to collect randomized controlled trials on use of PRP in AGA for a meta-analysis.

**Results:**

Ten trials with a total 555 treatment units were identified. The hair density in PRP group was significantly higher than control group [MD = 25.09, 95%CI: 9.03–41.15, *p* = 0.002], but there was no significant difference in hair diameter between two groups [SMD = 0.57, 95%CI: − 0.23 to 1.38, *p* = 0.16]. Subgroup analyses indicated that hair density was significantly higher among the male-only trials than in the mixed-sex samples (*p* = 0.02). In addition, neither the split-head design nor the year of publication affected hair density (*p* = 0.05, *p* = 0.06). However, hair density was significantly higher in trials with a sample size less than 30 (*p* = 0.0004).

**Conclusions:**

PRP treatment increased hair density in participants with AGA, but not hair diameter. In terms of hair density, PRP elicits stronger effects in male patients. There was a trend toward differed treatment effect by gender with PRP injection, which warrants further investigation. Especially in the case of female.

**Level of Evidence III:**

This journal requires that authors assign a level of evidence to each article. For a full description of these Evidence-Based Medicine ratings, please refer to the Table of Contents or the online Instructions to Authors https://www.springer.com/00266.

**Supplementary Information:**

The online version contains supplementary material available at 10.1007/s00266-023-03603-9.

## Introduction

Androgenetic alopecia (AGA), also known as male/ female pattern hair loss, is a common yet difficult-to-treat condition, with even mild cases having psychological impact on patients. Currently one of the most discussed treatment modalities for AGA is platelet-rich plasma (PRP) therapy. PRP has been advocated for promoting wound healing and tissue regeneration. Evidence from experimental studies indicates that PRP is associated with hair regrowth [1]. Therefore, PRP injection has been viewed as a potential strategy for alopecia. If PRP and its secretory factors were considered to contribute to the regulation of hair growth, PRP injection would be a safe, minimally invasive, easy and cost-effective method to treat AGA.

Current data examining the efficacy of PRP for treating AGA are inconsistent. Clinical randomized controlled trials and basic research have revealed a positive effect of PRP for AGA treatment [2–5]. Previous systemic review and clinical trial have showed limited benefit of PRP treatment for AGA [6, 7]. Interpretation of these reviews is difficult because the lack of a standard preparation of PRP and protocols for clinical treatment, which has been associated with the validity of inter-study comparisons [6, 8, 9]. Additionally, subjects in these reviews contained other types of baldness (e.g., alopecia areata) or the control group was not a placebo [3, 6].

Recently, additional trials assessing the effect of PRP on AGA have become available, which have remarkably enlarged the number of trial participants. Because of limitations of previous reviews, conflicting evidence, and availability of new data, we aimed to conduct a systematic review and meta-analysis of randomized controlled trials to evaluate the effect of PRP as a monotherapy for AGA.

## Material and Methods

### Guidance

This study was conducted in accordance with the Preferred Reporting Items for Systematic Reviews and Meta-Analysis (PRISMA) statement [10] and is registered under the PROSPERO (CRD42022357157).

### Inclusion Criteria

Inclusion criteria are as follows: (1) the type of study: randomized controlled trial (RCT); (2) types of interventions: the experimental groups were subjected regular use of PRP preparation, while the control groups received placebo saline; (3) published in English; (4) follow-up for at least 3 months.

### Exclusion Criteria

Exclusion criteria are as follows: (1) any study type other than RCT; (2) the experimental groups not treated with PRP alone or the control group not with placebo; (3) republished articles; (4) studies with less than 10 total subjects; (5) studies on non-AGA forms of alopecia; (6) articles containing incomplete information.

### Outcomes

The primary outcome was hair density (number of hairs/cm^2^). Secondary outcome was hair diameter.

### Search Strategy

One of the authors (MJL) performed the search of several databases: PubMed, Embase, Web of Science and Cochrane Library, from their inception to October 2022. Among them, the detailed retrieval strategies for PubMed were: ("Blood Platelets"[Mesh]) OR ("Platelet-Rich Plasma"[Mesh]) OR (PRP) OR (PRF) OR ("platelet plasma") OR ("platelet gel") OR ("platelet concentrate") OR ("buffy layer") OR ("platelet rich") AND ("Alopecia"[Mesh]) OR ("androgenic alopecia") OR ("androgenetic alopecia") OR ("hair loss") OR (baldness) OR ((androgen*). We extracted available data from published papers. To maximize the search for relevant articles, we scrutinized the reference lists of identified trials and systematic reviews.

### Study Selection

After removal of duplicates, two independent researchers screened the title and abstract of articles. If the information was not sufficient to access the eligibility, a full-text evaluation was performed. Studies that met the inclusion criteria were used as the meta-analysis in this work. Consensus was required for inclusion, and in case of disagreement, the studies were discussed by all the authors until a consensus was reached.

### Data Collection Process

Two independent researchers adopted a standard data extraction form to extract data from the included studies. When a trial mentioned an outcome of interest with different time points, we pooled data from the earliest time point. Disagreements were resolved by consensus.

### Assessment of Risk of Bias and Quality of Evidence

The quality of the included studies was evaluated based on the Cochrane risk of bias assessment tool for RCT [11].

### DATA Synthesis

We used Review Manager 5.3 software and STATA version 16.0 to calculate a 95% confidence interval (CI) for hair density, hair number, and hair diameter. Difference in those continuous variables was expressed as mean difference (MD)/standardized mean difference (SMD) and standard deviations (SD). To incorporate heterogeneity between papers, I2 values were tested. I2 values > 50% were considered highly heterogeneous and warranted investigation of the details that may cause heterogeneity. If the heterogeneity was low, we would use the fixed effect model for the meta-analysis. Conversely, we would use the random-effect model for the meta-analysis. Publication bias was assessed by the Egger’s test. We used the one by one exclusion method to perform sensitivity analysis. A *p* < 0.05 was considered significant difference between the experimental and control groups.

### Subgroup Analysis

We conducted several subgroup analysis to test interactions according to sex (male and both sexes); study design (split-head design and non-split-head design); year of publication (before or in 2017 and after 2017); number of participants (> 30 and ≤ 30).

## Results

### Eligible Studies and Study Characteristics

**Fig. 1:**
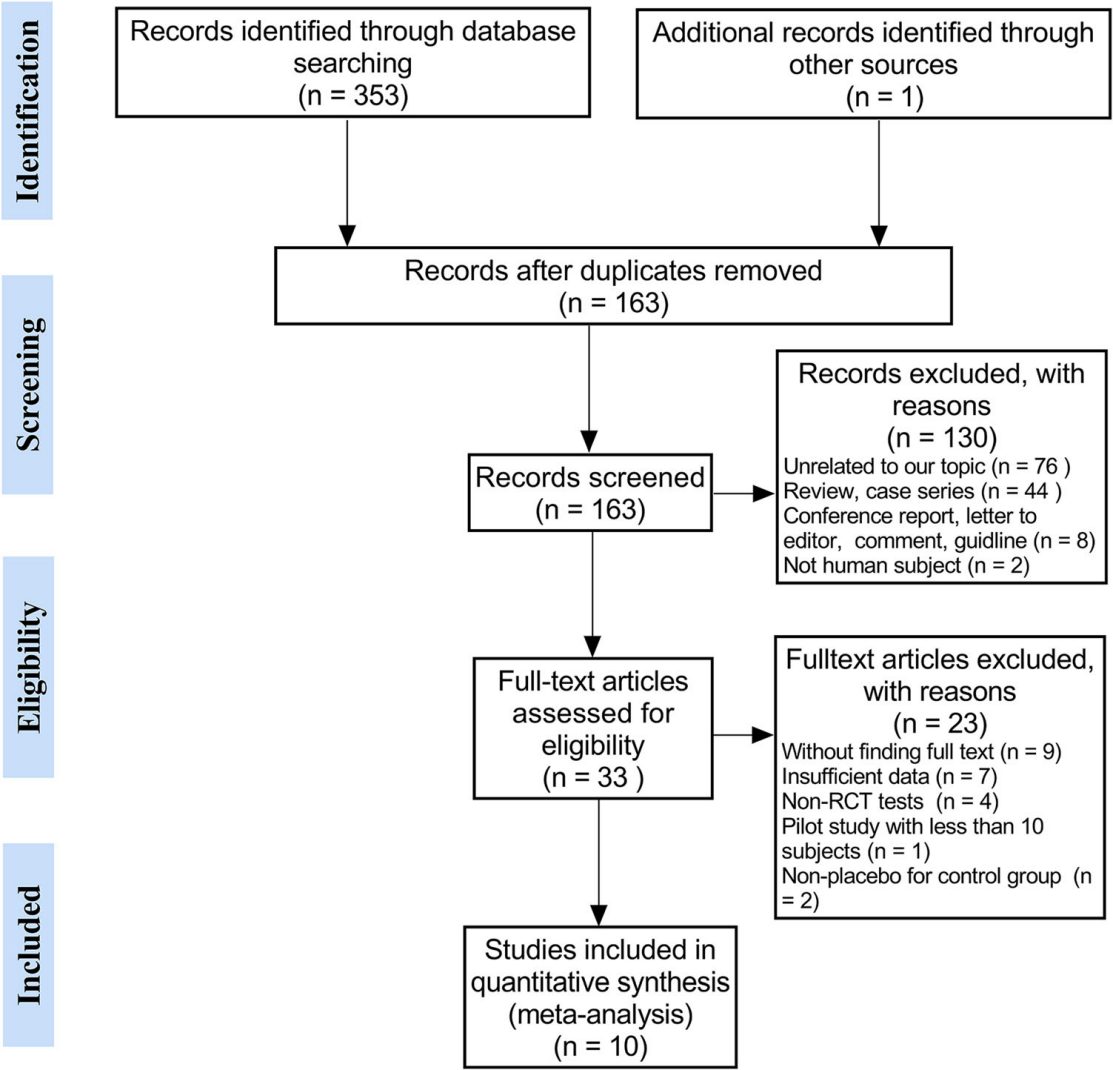
Study screening flow diagram

We initially identified 353 records and included 10 eligible trials [4, 7, 12–19] in the final meta-analysis (Fig. 1). 8 of these studies had a split head design or quasi-split head design, in which case the unit of analysis was each area of the scalp and not the individual. Table 1 presents a summary of included trials, and supplemental eTable 1 shows details of those trials. A total 555 treatment units from 318 participants were included in this study, with 275 treatment units in the PRP group and 280 treatment units in the control group.

**Table 1:** The information table for included studies

Author	Country	Study design	No. of subjects	Age	Males (AGA stage)/females (AGA stage)	Intervention group (*N*)	Control group (*N*)	Follow-up	Main conclusion
Alves [12].	Spain	RCT, split-head design	25	21–62	11 (II–V)/11(I–III)	PRP (22)	Placebo (22)	6 months	Application of PRP showed a positive effect on AGA
Cervelli [13]	Italy	RCT, study in the same patients	10	22–60	10 (II–IV)/0	PRP (10)	Placebo (10)	14 weeks	A significant increase in the hair density in the PRP treatment area
Dicle [15]	Turkey	RCT	30	21–48	25 (III–V)/0	PRP (10)	Placebo (15)	4 months	Application of PRP showed a positive effect on AGA in male
Gentile [17]	Italy	RCT, split-head design	23	19–63	23 (IIa–IV)/0	PRP (23)	Placebo (23)	14 weeks	A significant increase in the hair density in the PRP group
Shapiro [7]	USA	RCT, split-head design	35	18–58	18 (III–V)/17(I–II)	PRP (35)	Placebo (35)	6 months	No significant difference in both hair density and hair diameter between the two groups
Tawfik [18]	Egypt	RCT, split-head design	30	20–45	0/30 (I–III)	PRP (30)	Placebo (30)	6 months	A significant increase in both hair density and hair thickness in PRP group
Gentile [16]	Italy	RCT, split-head design	18	19–63	18 (II–III)/0	PRP (18)	Placebo (18)	3 months	A significant elevate in hair density for the A-PRP group
Qu [4].	China	RCT, split-head design	52	Not state	32 (II–V)/20 (I–III)	PRP (52)	Placebo (52)	6 months	The PRP treatment boosted hair regrowth in Chinese AGA patients
Toama [19]	Egypt	RCT	40	18–45	19 (Ι–V)/21 (Ι–II)	PRP (20)	Placebo (20)	6 months	A greater hair density in the PRP group
Chuah [14]	Singapore	RCT, split-head design	55	23–70	34 (III–VI)/21 (II–III)	PRP (50)	Placebo (50)	6 months	A significant increase in hair density only in male participants in PRP group

Supplemental eFigs. 1 and 2 show risk of bias. Six trials had a low risk of bias, three trials had an unclear risk, and one trial had a high risk of bias [20]. The quality of evidence for the primary outcome was high.

### Primary Outcome: Hair Density

**Fig. 2:**
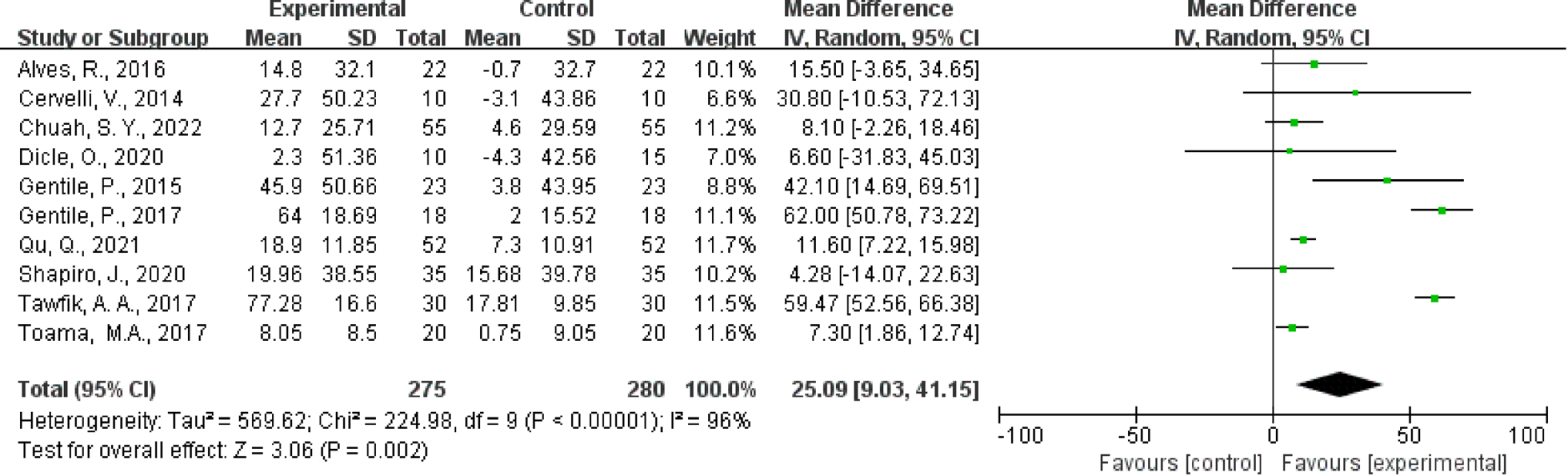
The pooled result of the hair density of 10 studies

All 10 trials reported hair density. This study found that the hair density was statistically significantly higher in PRP group than control group (mean difference 25.09 hairs/cm^2^, 95% confidence interval 9.03–41.15, *p* = 0.002, Fig. 2). Funnel plot analysis showed no asymmetry (eFig. 3); additionally the Egger test (*p* = 0.607) detected no significant small study effects. The meta-analysis results for hair density were robust in sensitivity analyses (eFig. 4).

Subgroup analyses found that hair density was significantly higher among the male-only trials than in the mixed-sex samples (*p* for interaction = 0.02, Table 2), although hair density was significantly increased in both groups. Subgroup analyses also found that hair density was significantly higher in trials with a sample size less than 30 (*p* for interaction = 0.0004, Table 2).

**Table 2:** Subgroup analysis of the effect of PRP on hair density.

Subgroup title	No of trials	No of participants/units	*I*^2^ (%)	Mean difference (95% CI)	*p* for interaction
Overall	10				
Sex					
Male	4	127	69	40.08 [15.53, 64.63]	0.02*
Male and female	5	368	0	9.73 [6.58, 12.88]	
Study design					
Split-head design	7	470	97	29.07 [7.84, 50.29]	0.052
Non-split-head design	2	65	0	7.29 [1.90, 12.67]	
No of participants					
≤ 30	6	231	82	40.64 [23.67, 57.61]	0.0004*
> 30	4	324	0	9.57 [6.38, 12.76]	
Year of publication					
Before or in 2017	6	246	97	36.49 [9.70, 63.28]	0.06
After 2017	4	309	96	10.71 [6.80, 14.63]	

*Statistically significant

### Secondary Outcome: Hair Diameter

**Fig. 3:**
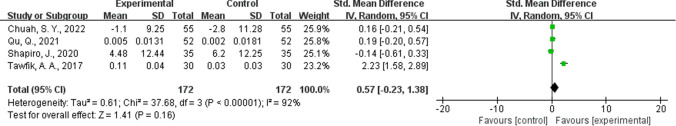
The pooled result of the hair diameter of 4 studies

There was no statistically significant difference in hair diameter between the PRP group and the control group (standardized mean difference 0.57, 95% confidence interval − 0.23 to 1.38, *p* = 0.16, Fig. 3).

## Discussion

In this meta-analysis of 10 randomized controlled trials with a total of 555 treatment units from 318 participants, PRP injection significantly increased hair density in subjects with AGA (mean difference 25.09 hairs/cm2, 95% confidence interval 9.03–41.15, *p* = 0.002), and this effect was more pronounced in male. However, the findings suggest that the use of PRP in AGA patients was not significantly associated with hair diameter (standardized mean difference 0.57, 95% confidence interval − 0.23 to 1.38, *p* = 0.16).

### Principal Findings and Comparison with Other Studies

The results of this study on hair density are largely consistent with previous systematic reviews. In 2021, a meta-analysis by Cruciani et al. that included 8 trials with a total of 308 treatment units found that PRP injections increased hair density (MD, 25.6 hairs/cm^2^; 95 % CI: 2.62–48.57; *p* = 0.03) [6]. A systematic review in 2022 also suggested PRP treatment increased hair density in analysis of 4 RCTs with a total of 182 units (MD, 26.77 hairs/cm^2^; 95 % CI: 9.90–43.63; *p* = 0.002) [8]. Besides, three systematic reviews published similar results [21–23]. Our findings on hair density are consistent with these reviews, but some of the methods used in these studies differ. The study by Cruciani et al. included one trial in patients with alopecia areata (AA), which was excluded in our study. In addition, these reviews were also limited by the number of trials (n≤3), combined analysis of experimental (RCTs and non-RCTs) and observational studies (retrospective, prospective and cohort study), and mixed interventions (PRP plus other drugs). Compared with these reviews, we included only RCTs of PRP monotherapy for AGA compared with placebo. Moreover, this study additionally included 4 RCTs published after 2020, so that the more recent trials accounted for 55.7% (309/555) of the total number of treatment units. More importantly, our study found that male subjects treated with PRP had superior outcomes in terms of hair density. This will further be elaborated later in the discussion.

In contrast to the results for hair density, this study did not find evidence to suggest that PRP treatment increased hair diameter. The results of this meta-analysis on hair diameter differ from two previous systematic reviews [8, 24]. A systematic review in 2022 found that PRP injection increased hair diameter in analysis of two trials with a total of 220 units (MD = 28.77, 95% CI: 12.52–45.03; *p* = 0.0005)[8]. In 2020, a meta-analysis by Gupta et al. that included 9 trials with a total of 218 participants also suggested a significant effect on hair diameter in both men and women (MD = 6.66, 95% CI: 2.37–10.95, *p* = 0.002; MD = 31.22, 95% CI: 7.52–54.91, *p* = 0.01) [24]. The previous reviews probably reached more optimistic conclusions as a result of different study selection criteria and newly published studies. In that 2022 study, one of the two trials used another PRP treatment protocol control rather than placebo. Compared to the study by Gupta et al., we excluded non-randomized controlled trials, raising the evidence level. Moreover, we added three newly published trials totalling approximately 284 units from 142 participants, greatly increasing the evidence base available for analysis.

However, the results of this study on hair diameter were in conflict with the expected results. The possible reasons for this might include the following two reasons. Firstly, it is possible that a 6 month follow-up was not long enough to see an effect on hair diameter. Secondly, scalp injections of saline in the control group, where growth factors and cytokines were perhaps released by the wound healing process secondary to scalp injections, similar to microneedling treatment.

An important finding from our subgroup analysis was that the effect of PRP differs for male and mixed-sex participants. We found that hair density was significantly higher among male participant with AGA than in mixed-sex subjects suffering from AGA; despite hair density significantly increased in both subgroups compared to the control group. The potentially gender-specific effects of PRP for AGA might be associated with the clinical features. In AGA, the progression differs markedly between genders. For male, hair lost in defined patterns and often result in complete baldness. For female, characterized by diffuse thinning, and rarely leads to complete baldness. From the clinical presentation, male patients have more hair density reduction at the onset, which may be the reason why hair density recovered more significant after PRP treatment than female patients. This speculation was similar to the results of Gupta et al. [24]. Their review found that PRP significantly increased hair density in male (MD, 25.83 hairs/cm^2^; 95% CI: 15.48–36.17, *p* < 0.00001), but not in female (MD, 43.54 hairs/cm^2^; 95% CI: − 1.35 to 88.43, *p* = 0.06). In 2022, the Chuah trial [14] also reported similar results, but suggested that the relatively fewer female participants might influence some of the results. However, there is a lack of RCTs on female AGA and few studies on AGA in male and female separately, which can have an impact on the results. Therefore, the effect of PRP on hair density by gender requires additional evidence, preferably gathered by future large randomized controlled trials.

A further important finding from our subgroup analysis was that hair density was statistically significantly higher in trials with lager participants numbers. However, no previous reports were found in the literature regarding the association between sample size and the effect of PRP. It is important to note that small size studies may overestimate the effect of the PRP treatment.

### Strengths and Limitations

This study has several methodological strengths. We followed the recommendations outlined of the Cochrane Collaboration and PRISMA statement. This meta-analysis also was registered in advance on PROSPERO. In addition, this study included a rigorous assessment of the quality of evidence using RevMan (the quality for the primary outcome was high).

Study has important limitations. This systematic and meta-analysis was based solely on published trials that PRP as a monotherapy for AGA. However, there was an overall predominance of male participants (61%), which suggests that substantial selection reporting was likely. Also, this study was not able to assess the long-term (> 12 months) effects of PRP therapy due to the lack of enough time points in the trials evaluated.

The PRP preparation in included trials varied. Our study could not accurately compare equivalent PRP dose in the included trials because they all had different standardization of PRP production (platelet concentration) and protocols (centrifugation details, with or without activator, etc.). This might be one of the reasons why this study did not determine an effective PRP preparation. Furthermore, the platelet basal level and response ability vary among different individuals, which also affect the PRP clinical effect. These limitations and uncertainties associated with PRP preparation and individual variability of platelet warrant further investigation.

Heterogeneity in meta-analyses should always be paid attention to. In this study, important statistical heterogeneity was found in the main analysis. Heterogeneity anticipated was mainly the small sample sizes of some included studies, gender of the patients, nationalities and races of patients, grade of AGA, the different PRP preparation treatment regimes. However, no significant publication bias was found by funnel plots and Eggers tests, and the sensitivity test indicated that our conclusion was reliable.

## Conclusions

Overall, PRP treatment increased hair density in participants with AGA, but not hair diameter. In terms of hair density, PRP elicits stronger effects in male patients. There was a trend toward differed treatment effect by gender with PRP injection, which warrants further investigation.

### Supplementary Information

Below is the link to the electronic supplementary material.

Supplementary file1 (DOCX 49 kb)

Supplementary file2 (TIF 656 kb)

Supplementary file3 (TIF 173 kb)

Supplementary file4 (TIF 2478 kb)

Supplementary file5 (TIF 1022 kb)

Supplementary file6 (TIF 356 kb)

Supplementary file7 (TIF 1189 kb)

Supplementary file8 (TIF 370 kb)

Supplementary file9 (TIF 370 kb)

Supplementary file10 (TIF 1022 kb)

Supplementary file11 (TIF 172 kb)

Supplementary file12 (TIF 2478 kb)

## Data Availability

All data generated or analyzed during this study are included in the article and Supplemental material file. Future enquiries can be directed to the corresponding author.
