# Supplementary material for: Effectiveness of Platelet-Rich Plasma in the Treatment of Androgenic Alopecia: A Meta-Analysis
Source: Aesthetic Plast Surg. 2023 Aug 29;48(5):977–84. doi: 10.1007/s00266-023-03603-9 (PMC10980625; doi:10.1007/s00266-023-03603-9)
Supplement: Supplementary file 1 — Supplementary file1 (DOCX 49 kb) [file 266_2023_3603_MOESM1_ESM.docx]

**Supplemental e-material**

eFig.1 The Cochrane risk bias summary.

eFig.2 The Cochrane risk bias graph.

eFig.3 Funnel plot of hair density.

eFig.4 Sensitivity analysis for hair density.

eFig.5 The pooled result of the hair density from gender.

eFig.6 The pooled result of the hair density from study design.

eFig.7 The pooled result of the hair density from the year of publication.

eFig.8 The pooled result of the hair density from sample size.

eFig.9 Runnel plot of hair diameter.

eFig.10 Sensitivity analysis for hair diameter.

eTable 1 The PRP preparation and treatment protocols.
