# Supplementary figures and images for: Effectiveness of Platelet-Rich Plasma in the Treatment of Androgenic Alopecia: A Meta-Analysis
Source: Aesthetic Plast Surg. 2023 Aug 29;48(5):977–84. doi: 10.1007/s00266-023-03603-9 (PMC10980625; doi:10.1007/s00266-023-03603-9)

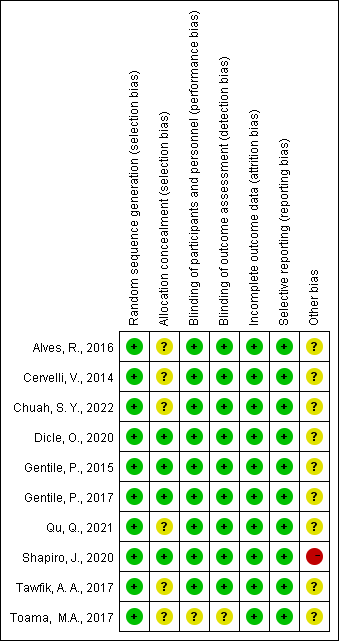

Supplement: Supplementary file 2 — Supplementary file2 (TIF 656 kb) [file 266_2023_3603_MOESM2_ESM.tif]

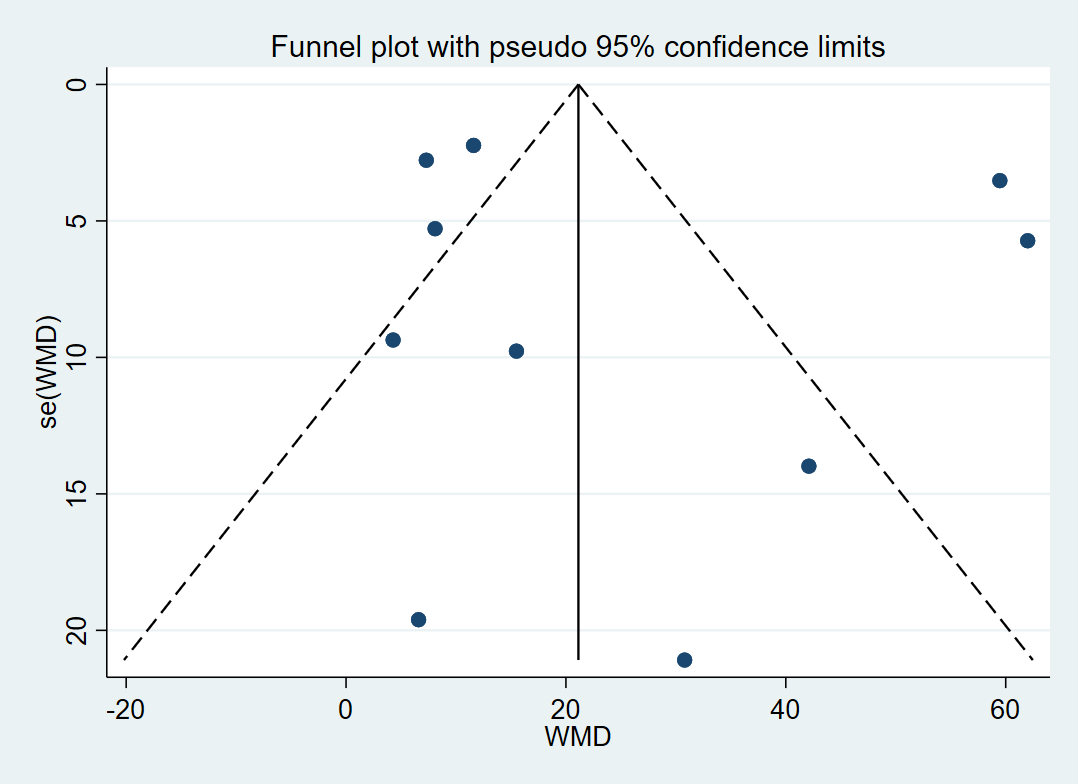

Supplement: Supplementary file 4 — Supplementary file4 (TIF 2478 kb) [file 266_2023_3603_MOESM4_ESM.tif]

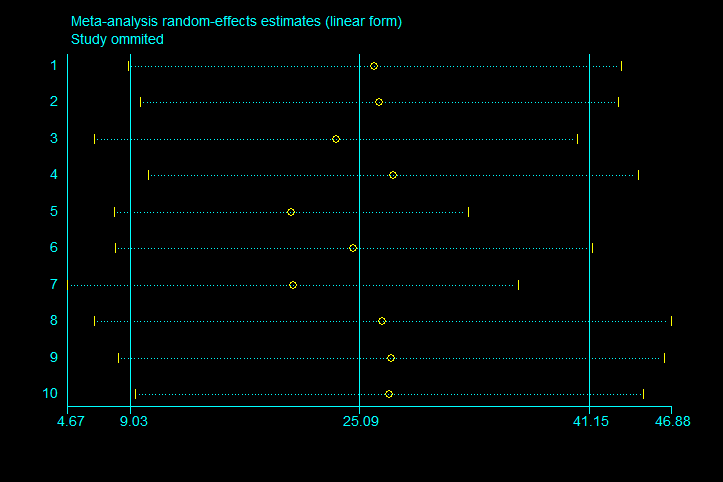

Supplement: Supplementary file 5 — Supplementary file5 (TIF 1022 kb) [file 266_2023_3603_MOESM5_ESM.tif]

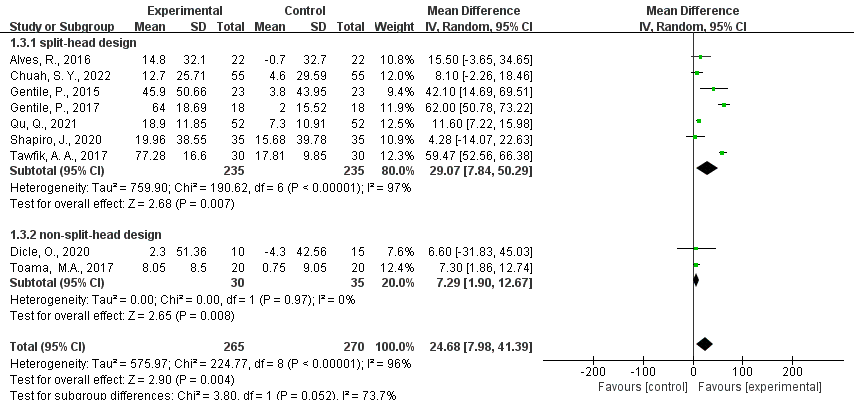

Supplement: Supplementary file 7 — Supplementary file7 (TIF 1189 kb) [file 266_2023_3603_MOESM7_ESM.tif]

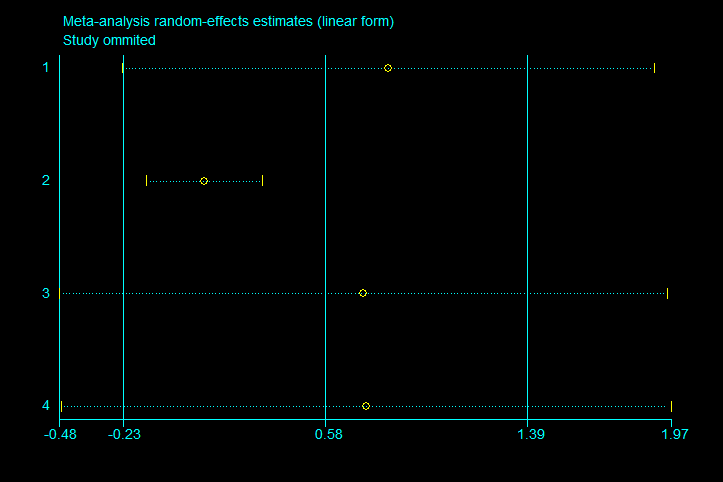

Supplement: Supplementary file 10 — Supplementary file10 (TIF 1022 kb) [file 266_2023_3603_MOESM10_ESM.tif]

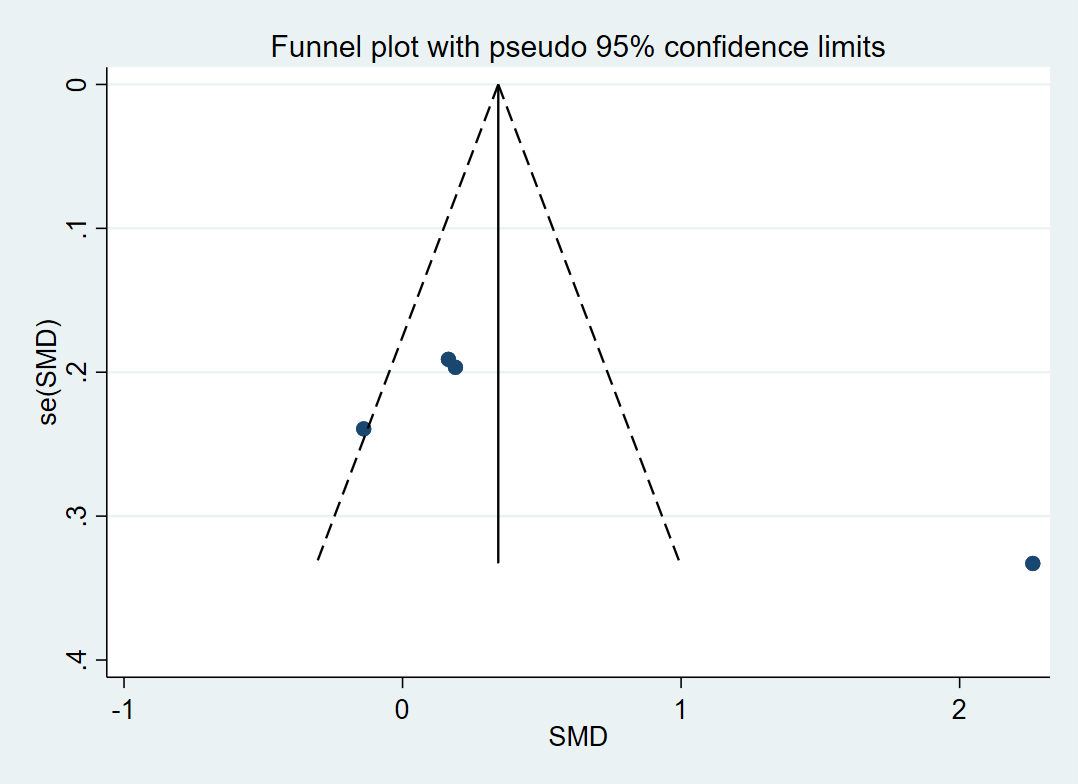

Supplement: Supplementary file 12 — Supplementary file12 (TIF 2478 kb) [file 266_2023_3603_MOESM12_ESM.tif]
